# Simulation of synaptic short-term plasticity using Ba(CF_3_SO_3_)_2_-doped polyethylene oxide electrolyte film

**DOI:** 10.1038/srep18915

**Published:** 2016-01-07

**Authors:** C. T. Chang, F. Zeng, X. J. Li, W. S. Dong, S. H. Lu, S. Gao, F. Pan

**Affiliations:** 1 Key Laboratory of Advanced Materials (MOE), School of Materials Science and Engineering, Tsinghua University, Beijing, 100084, People’s Republic of China; 2 Centre for Brain Inspired Computing Research (CBICR), Tsinghua University, Beijing 100084, People’s Republic of China

## Abstract

The simulation of synaptic plasticity using new materials is critical in the study of brain-inspired computing. Devices composed of Ba(CF_3_SO_3_)_2_-doped polyethylene oxide (PEO) electrolyte film were fabricated and with pulse responses found to resemble the synaptic short-term plasticity (STP) of both short-term depression (STD) and short-term facilitation (STF) synapses. The values of the charge and discharge peaks of the pulse responses did not vary with input number when the pulse frequency was sufficiently low(~1 Hz). However, when the frequency was increased, the charge and discharge peaks decreased and increased, respectively, in gradual trends and approached stable values with respect to the input number. These stable values varied with the input frequency, which resulted in the depressed and potentiated weight modifications of the charge and discharge peaks, respectively. These electrical properties simulated the high and low band-pass filtering effects of STD and STF, respectively. The simulations were consistent with biological results and the corresponding biological parameters were successfully extracted. The study verified the feasibility of using organic electrolytes to mimic STP.

Brain-like computation is studied worldwide to suppress the limitations of the Moore Principle and to construct novel computing technology^1^,^2^,^3^. The determination of elements possessing synapse-like behaviours is a powerful and effective route to realize this target. Researchers have found many materials and devices that mimic synaptic plasticity, including conventional learning protocols, such as the Hebbian learning rule, spike-rate-dependent plasticity (SRDP), spike-timing-dependent plasticity (STDP), long-term plasticity (LTP) and short-term plasticity (STP)^4^,^5^,^6^,^7^,^8^,^9^,^10^,^11^,^12^,^13^,^14^,^15^,^16^,^17^,^18^,^19^. However, a material system that can act as the elemental unit of the synapse in artificial neuromorphic circuits has not yet been found. Thus, much research has investigated novel materials with behaviours closely approximating those of bio-synapses.

Recently, systems composed of organic materials have captured the attention of researchers because they possess physical and chemical properties comparable with those of biological systems. In a nanoparticle-doped organic field effect transistor (OFET), the charge/discharge mechanism of a nanoparticle-doped semiconducting polymer^8^ was used to simulate excitatory post-synaptic current (EPSC)-like responses and STP-like^15^,^16^,^18^ behaviour under a frequency-dependent pulse mode. Ionic migration in ionic/electronic hybrid systems was first used by Lai *et al.* to obtain EPSC-like responses and STDP learning protocols^10^. In addition, a double-layer device composed of a semiconducting polymer and a Li-doped electrolyte exhibited frequency selectivity, similar to that of bio-synapses dependent on surface ionic migration; the device responded with depression to low-frequency stimulation (LFS) and with potentiation to high-frequency stimulation (HFS)^20^. Since the plasticity of bio-synapses directly involves the ionic flux crossing the cell membrane, the ionic kinetics in either organic materials or interfaces thereof suggest great versatility in simulating synaptic plasticity^9^,^20^,^21^,^22^,^23^,^24^. The selection of certain dopant species in artificial organic materials may provide a system that approximates the behaviours and timing constants of real bio-synapses.

Among many synaptic plasticity properties^15^,^16^,^17^,^23^, STP^15^,^16^,^18^, which acts as a filter system in biological signal transmission, is crucial in the support of nerve operations^16^. However, studies using memristive systems have not focused on this property, with the exception of that involving a nanoparticle-doped semiconducting polymer^8^ and another using solid-state TiO_2_ memristor^19^. In the forms and definitions of high band-pass filtering short-term depression (STD)^16^, low band-pass filtering short-term facilitation (STF), and the intermediate state between these conditions, STP is characterized by the weight-changed EPSCs^25^,^26^ and controlled by the number of effective neurotransmitter release sites and the concentration of 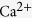 in the pre-synapse^16^,^18^,^27^. In addition, STP gradually varies with frequency, weight change history, and a persistence time of hundreds of milliseconds^15^. Unlike long-term plasticity (LTP)^17^, which has been realized in several nonvolatile memristor devices^28^, STP has rarely been achieved in devices, despite its development in several biological computational models^27^,^29^.

In this study, we provided a single-layer salt-doped organic electrolyte film device with polyethylene oxide (PEO), which could be fabricated easily using large-scale engineering processes. The film served as the matrix, while Ba(CF_3_SO_3_)_2_ (barium trifluoromethanesulfonate) was used as the salt to simulate the synaptic operating process and the typical band-pass filtering characteristics of STP. Ion-doped organic electrolytes are uncommon in the field of electronics, despite the obvious advantages of easily alternating and mixing ions. The Ba(CF_3_SO_3_)_2_-doped PEO responded gradually to saturation with pulse number, and the weight modifications of the charging peaks differed from those of the discharging peaks, indicating distinct band-pass filtering effects. A biological EPSC model of STP proposed in neuroscience^27^ was adopted to analyse the pulse responses of the device; characteristic parameters were extracted that were comparable with those of bio-synapses.

## Methods

PEO (molecule weight = 100000) and Ba(CF_3_SO_3_)_2_ were purchased from Sigma-Aldrich Co. Ltd. and used as without further purification. PEO was dissolved in H_2_O to form a 0.4wt% solution with Ba(CF_3_SO_3_)_2_ added in the molar ratio of 1:32 with the PEO monomer^20^,^21^. After dissolution, 3 μL of the Ba(CF_3_SO_3_)_2_-PEOsolution was drop-casted on a Pt-deposited Si substrate and baked at 100 °C for 20 min before cooling to room temperature in a N_2_-filled glove box. Finally, a 70-nm-thick layer of 300-μm-diameter Pt electrodes were deposited on the substrate by electron beam deposition. The thickness of the Ba(CF_3_SO_3_)_2_-doped PEO film was about 0.6 μm (Fig. S1). The electrical characteristics were examined by a semiconductor device analyser (Agilent B1500A) and an arbitrary function generator (Agilent B1530). The direct voltage-current (DC) properties were studied by loading a circular single bias of 0 V → 2 V → 0 V and a double bias of 0 V → 2 V → 0 V → −2 V → 0 V under sweep rates of 1, 10, 50, and 100 V/s. The pulse responses were studied by loading a train of rectangular pulses with 0.5 Vamplitude, 5 ms pulse width, and varied intervals or frequency. Raman spectra of the pure PEO film and the Ba(CF_3_SO_3_)_2_-doped PEO electrolyte film were obtained by an HR-800 Raman system with a resolution of 1 cm^−1^.The excitation source was a 532-nm He-Ne laser. Infrared spectra were recorded with a Vertex 70V Fourier transform infrared (FT-IR) spectrometer at a resolution of 0.4 cm^−1^.

## Results and Discussions

### Structural Characterization

**Figure 1:**
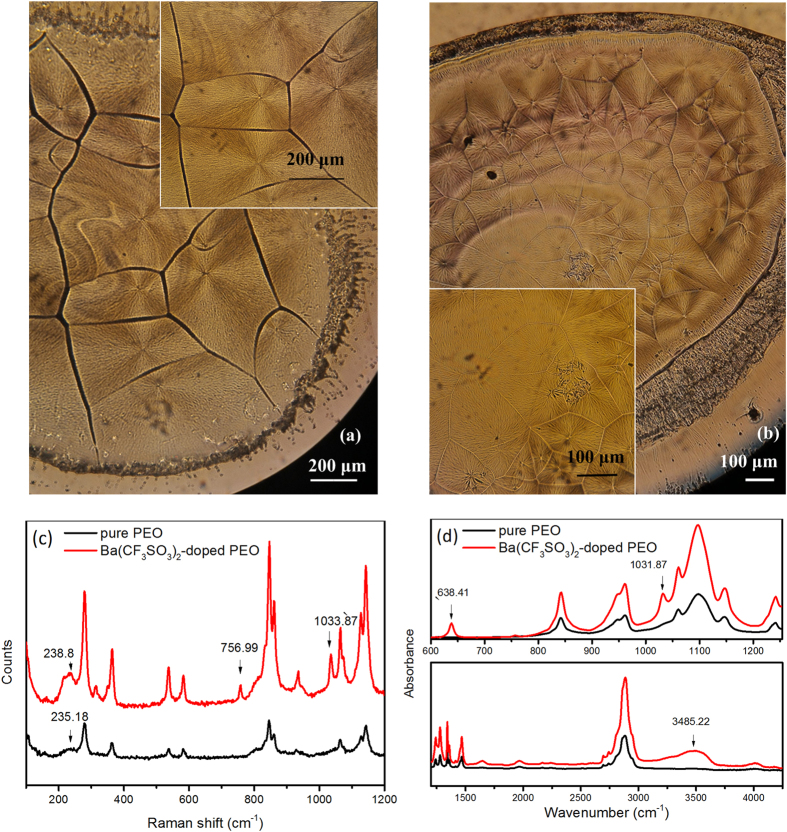
Structural characterization of the electrolyte films. Photomicrographs of (**a**) pure PEO film and (**b**) Ba(CF_3_SO_3_)_2_-doped PEO film. (**c**) Raman spectra for pure PEO and Ba(CF_3_SO_3_)_2_-doped PEO films. (**d**) FT-IR spectra for pure PEO and Ba(CF_3_SO_3_)_2_-doped PEO films.

The ionic conductivity of PEO-based electrolytes is attributed to ionic migration through the amorphous phase of the PEO matrix, with free cations traversing the polymer backbones through complexation with polar groups and the movement of free anions^30^. Therefore, the amorphous phase of the PEO matrix, cation-polymer complexation, and free ions are vital for the conductivity of the electrolyte. In the photomicrographs shown in Fig. 1, the pure PEO film (Fig. 1a) presents a spherulitic morphology with dendritic lamellae, which indicates a normal polycrystalline structure bridged by amorphous regions^31^. The Ba(CF_3_SO_3_)_2_-doped PEO film (Fig. 1b) has an analogous spherulitic morphology with more obscure dendritic lamellae patterns and grain boundaries. This reveals a decrease in lamellae and an increase in amorphous regions compared with the structure of the pure PEO film, which is helpful for the ionic conductivity of the doped polymer.

We examined the cation-polymer interactions by spectrally monitoring the PEO backbone-related bands corresponding to C-O-C, CH_2_, CO, CC, and OH groups, and examined the ion-ion associations by monitoring the *υ*_*s*_(SO_3_) and *δ*_*s*_(CF_3_) bands^30^,^32^ based on the Raman spectra and the IR spectra of the pure PEO and Ba(CF_3_SO_3_)_2_-doped PEO films (Fig. 1c,d). based on the Raman and IR spectra of the pure PEO and Ba(CF_3_SO_3_)_2_-doped PEO films (Fig. 1c,d). In the Raman spectra (Fig. 1c), the backbone-related bands are slightly shifted and broadened in the spectrum of the doped PEO film compared to the peaks of that of the pure one. In particular, the shift of the D-LAM (disorder-longitudinal acoustic mode) peak at 235.18 cm^−1^ to 238.8cm^−1^ reveals that the cations do interact with the polymer backbones, causing conformational and amorphicity changes^30^. Additional bands at 1033.87cm^−1^ and 756.99 cm^−1^ exhibit the existence of free ions of *υ*_*s*_(SO_3_) and ion pairs of *δ*_*s*_(CF_3_), respectively^32^. In the IR spectrum of the doped PEO film, compared with that of the unmodified film (Fig. 1d), the band at 1031.87 cm^−1^ shows the existence of *υ*_*s*_(SO_3_) free ions^32^, while the 3485.22 cm^−1^ broad band correlating to OH stretching vibrations indicates cation-polymer interactions^33^,^34^. The 638.41 cm^−1^ band in the finger print region displays a pattern unique to the structure^33^,^34^. According to this structural analysis, the conductivity of the Ba(CF_3_SO_3_)_2_-doped PEO film results from ionic migration through cation-polymer interactions and partial free anions, which constrain the electrical transmission speed and decrease the speed of electric migration. Thus, the macroscopic electrical properties depend on the mode and history of the external field, thus causing the appearance of synaptic plasticity.

### Electrical characteristics: DC mode and pulse mode

**Figure 2:**
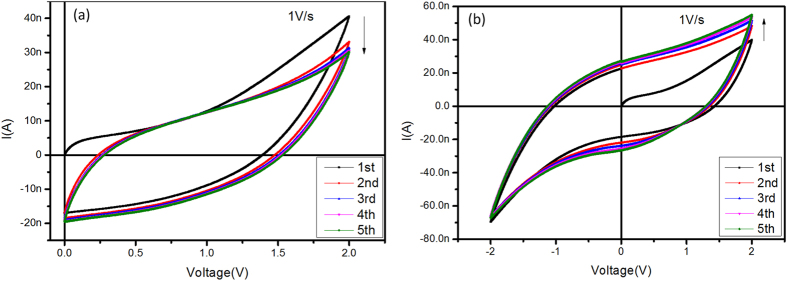
The electrical characterization of the doped PEO film in DC mode. (**a**) Successive single biases of 0 V → 2 V → 0 V … were applied to the device for five cycles. (**b**) Successive double biases of 0 V → 2 V → 0 V → −2 V → 0 V … were applied to the device for five cycles.

In the DC sweeping mode, the current routes varying along the external field (*E*_*ex*_) bias cycles do not coincide, and then form mismatching hysteresis loops (Figs. 2a and S2). This is similar to results with inorganic memristors^35^. When loading single biases (Fig. 2a), the loop sequences shift sequentially downward. Along the loop, the current increases with the forward bias, before decreasing with the backward bias through a lower current path. The current path then enters the region of negatively charged currents at a certain voltage threshold, reaching a maximum at 0V. This phenomenon demonstrates the migration and accumulation of Ba^2+^ and CF_3_SO_3_^−^ ions at the electrode/electrolyte interface during the forward biases, simultaneously forming polarization layers with an inverted internal field (*E*_*in*_)^36^. The *E*_*in*_ then gradually offsets the *E*_*ex*_, resulting in negative currents that balance the ionic distribution. As the most non-uniform ionic distribution occurs at 0 V (with the maximum negative current and *E*_*in*_), the *E*_*ex*_ of the following bias cycle should overcome the reserved *E*_*in*_. Therefore, the hysteresis loops shift down and approached a stable-loop state of ionic kinetic balance. When loading double biases (Fig. 2b), the consecutive hysteresis loops are gradually expanded, suggesting that the negative bias not only balances the non-uniform polarization layers of Ba^2+^and CF_3_SO_3_^−^ but also further creates an inverted polarized field providing an *E*_*in*_ with the same orientation as the positive bias. The narrower hysteresis loops generated at lower voltage sweeping rates suggests a stronger restrictive *E*_*in*_ of the polarization layers, caused by the more effective response of the Ba^2+^ and CF_3_SO_3_^−^ ions to the *E*_*ex*_ (Fig. S2). Overall, the behaviour in DC mode confirms the ionic kinetics of the PEO-based electrolyte.

**Figure 3:**
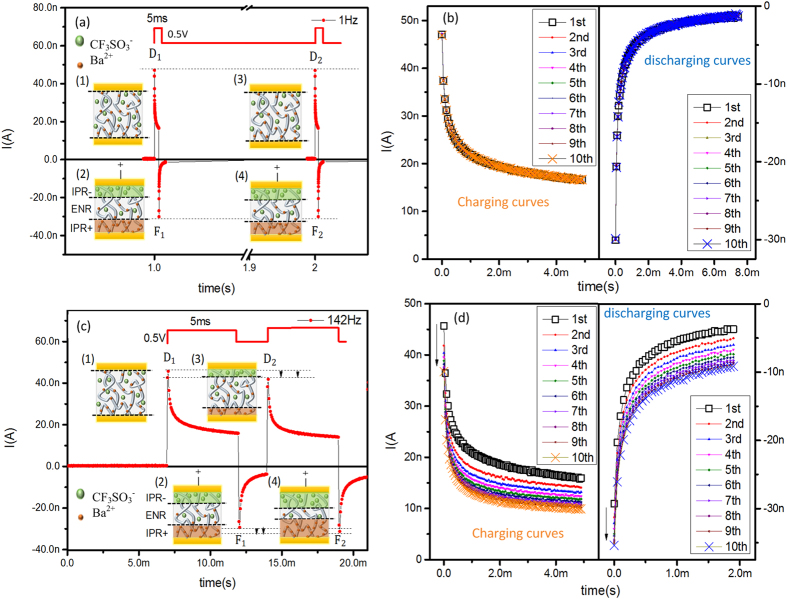
The electrical characterization of the doped PEO film in response to pulsed loads. Rectangular pulses are applied with amplitude of 0.5 V and duration of 5 ms. A train of pulses is loaded to test frequency response of the film. D_*i*_ and F_*i*_ are charging and discharging peaks for the *i*^*th*^ pulse, respectively. (**a**) Under 1 Hz pulse frequency, D_*i*_ and F_*i*_ sequences remain constant with the pulse number. (1) The initial electric neutral region (ENR) results in D_1_. (2) The ionic polar region (IPR) induces F_1_. (3) The fully-recovered ENR causes D_2_ = D_1_. (4) The same-sized IPR induces F_2_ = F_1_. (**b**) The charging and discharging curves of the first ten pulse responses under 1 Hz. (**c**) Under 142 Hz pulse frequency (HFS), D_*i*_ decreased and F_*i*_ increased with increasing *i*. (1) The initial electric natural region (ENR) results in D_1_. (2) The ionic polar region (IPR) induces F_1_. (3) The partially-recovered ENR results in D_2_ < D_1_. (4) The larger-sized IPR formed by the partially-decayed ENR generates F_2_ > F_1_. (**d**) The charging and discharging curves of the first ten pulse responses at 142 Hz.

The electrical characterization with DC mode suggested that the device responded depending on the loading history. It shares a resemblance with the signal handling of informatics and plasticity studies in neuroscience, which were tested under rectangular pulsing modes. Thus, we adopted the same testing approach to examine the exact comparability with our device. In Fig. 3a of LFS pulse mode sketches, a charging peak (D_1_) appears at the pulse front and decays gradually to a stable current value within the pulse duration. A discharging peak (F_1_) appears at the pulse end and decays gradually to zero. Notably, D_2_ and F_2_ in Fig. 3a are identical to D_1_ and F_1_, respectively, as are the charging and discharging curves (Fig. 3b). This demonstrates the state restoration cycles between loading pulses. However, when the interval between consecutive pulses is sufficiently short, as in the HFS shown in Fig. 3c, F_1_ does not decay to zero at the next pulse front; the following pulse period begins with a modified state of decreased D_2_ and increased F_2_, as were the charging and discharging curves (Fig. 3d). F_1_ required a full decay period of 0.52 s to provide a restored state of zero for the following load pulses. In addition, the non-volatile effect does not appear, even when the load pulse frequency approaches the limit of 142 Hz (Fig. S3).

In comparing general capacitor and resistor, the response form of our device was similar to that of the R//C device, but with an additional state modification phenomenon in HFS that was not found in the general R//C device (Fig. S4).When searching for connections with capacitance effects, we fitted the charging/discharging curves and found them to match the R//C charging/discharging equations well, with the modification of two capacitance terms of the form A*exp(−t/τ)(Table 1). The time constants τ_1_ and τ_2_ were nearly equal between the 1 Hz pulse response and the first response at 142 Hz. However, they both decreased with increasing pulse number at 142 Hz in the charging curves, but increased in the discharging curves. This implied great influences of the ion pair kinetics (Ba^2+^ and CF_3_SO_3_^−^) on the capacitance properties of the device, rather than the electronic conductivity with fast response rates. The hysteretic ionic conductivity may be the core of the state modification phenomenon.

By combining the ionic kinetics results obtained under DC mode, the conductivity and ionic distributions of our device seemed to experience a process under pulsing mode as follows. Initially, the ionic distribution in the Ba(CF_3_SO_3_)_2_-doped PEO was uniform, creating an electric neutral region (ENR) without polarity (states labelled (1) in Fig. 3a,c). When a train of rectangular pulses was loaded, D_1_ occurred because of the *E*_*ex*_-triggered carrier injection. Meanwhile, the directional migrations of free cations and anions occurred, forming an ionic polar region (IPR) along with *E*_*in*_ (states labelled (2) in Fig. 3a,c)^36^. The *E*_*in*_ partially screened the *E*_*ex*_-triggered ion migration, causing the current decay throughout the pulse duration. As the pulse ended, the IPR-related *E*_*in*_ triggered the inverse discharging peak F_1_ and the back-diffusing ionic flux. The latter was weakened by the gradual IPR decay.

The abovementioned back-diffusing ionic flux varied depending on the pulse frequency. In LFS (Fig. 3b), both cations and anions in the PEO matrix fully recovered to the initial state during the pulse interval, resulting in fully recovered ENR and fully decayed IPR (state labelled (3) in Fig. 3a). Thus, the instant carrier injection D_2_ evoked by the *E*_*ex*_ of the second pulse was equal to D_1_. Meanwhile, F_2_was equal to F_1_ because the *E*_*ex*_-triggered ionic relaxation created an equal-sized IPR as that formed in state (2) (state labelled (4) in Fig. 3a). When the frequency was increased (Fig. 3c), the ionic distribution could not fully revert to the initial state, because the pulse interval was insufficient. This generated a partially-recovered ENR and a residual IPR (state (3) in Fig. 3c). Thus, D_2_ was a counteractive result of the second *E*_*ex*_ and the *E*_*in*_ from the residual IPR, causing D_2_ < D_1_. In addition, within the second pulse duration, the IPR was enhanced by the *E*_*ex*_-triggered ionic concentration polarization, thus becoming wider than that evoking F_1_, so F_2_ > F_1_ (state (4) in Fig. 3c).

**Figure 4:**
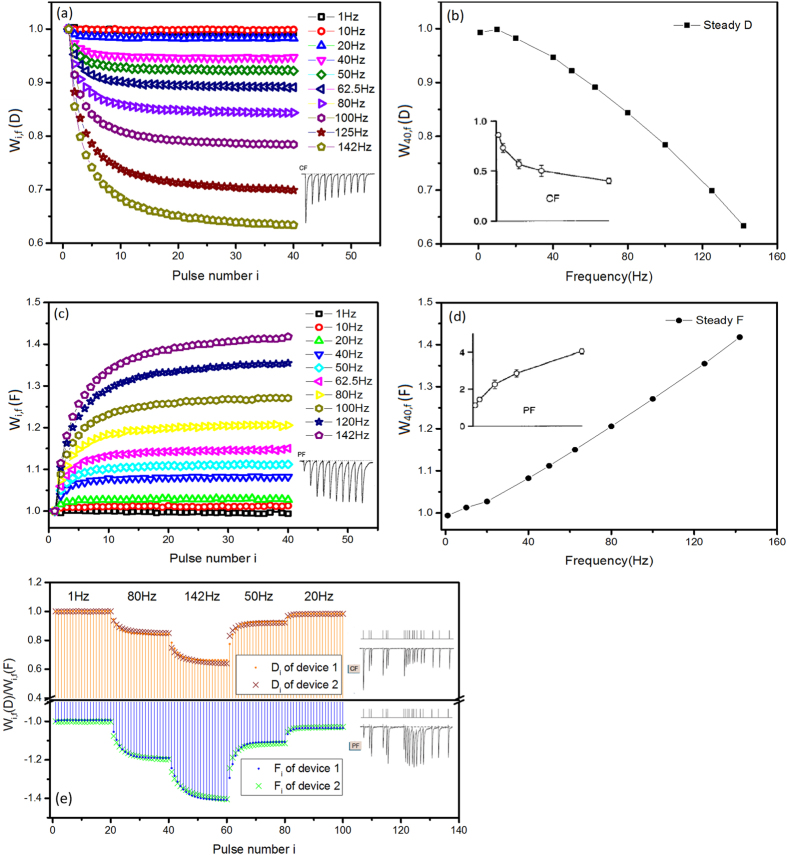
Weight modifications of D_*i*_ and F_*i*_ sequences under the pulse mode. (**a**) The weight modifications of D_*i*_ *vs* pulse number at various frequencies: *W*_*i,f*_* (D)* = *I(D*_*i,f*_*)/I(D*_*1,f*_). Inset shows EPSCs of climbing fibre (CF) under HFS, which decreases with increasing pulse number^27^. (**b**) The weight modifications of D_*i*_*vs.* frequency after a train of 40 pulses is loaded; inset shows those of CF:^27^ *W*_*,n,f*_ *(D)* = *I(D*_*n,f*_*)/I(D*_*1,f*_*), n* = *40*. (**c**) The weight modifications of F_*i*_*vs.* pulse number at various frequencies: *W*_*i,f*_* (F)* = *I(F*_*i,f*_*)/I(F*_*1,f*_). Inset shows EPSCs of parallel fibre (PF) under HFS^27^. (**d**) Weight modifications of F_*i*_*vs.* frequency after a train of 40 pulses is loaded; the inset shows those of PF:^27^*W*_*n,f*_* (F)* = *I(F*_*n,f*_*)/I(F*_*1,f*_*), n* = *40*. (**e**) A set of D_*i*_ and F_*i*_ generated by a pulse package containing several frequencies. The results of the two different single devices were tested during the same testing period and showed similar weight modification levels. Insets show EPSC variations of CF and PF in a pulse package containing several frequencies^16^.

The results in Fig. 3 also suggested that both the charging and discharging peaks D_*i*_ and F_*i*_ are functions of the pulse number *i* and the pulse frequency. We can calculate the weight modifications with the formulae *W*_*i,f*_ *(D)* = *I(D*_*i,f*_*)/I(D*_*1,f*_) and *W*_*i,f*_*(F)* = *I(F*_*i,f*_*)/I(F*_*1,f*_), respectively. Here we regard the first pulse responses of either *I(D*_*1,f*_) or *I(F*_*1,f*_) as the baseline values, because they are unchanged after sufficient intervals between two single pulses. Figure 4 shows the weight modifications of D_*i*_ and F_*i*_ sequences under the pulse mode. The values of D_*i*_ and F_*i*_ monotonically decreased and increased, respectively, with pulse number. When the frequency was lower than 80 Hz, the values of D_*i*_ and F_*i*_ stabilized after a train of 40 pulses. When the frequency exceeded 80 Hz, they were not saturated when the pulse train ended. Notably, the weight amplitude of D_*i*_does not match that of F_*i*_. This might suggest the potential for bi-directional signal transmission, although related works were not found among the available neuroscience references.

Interestingly, we found that the weight modifications of D_i_ and F_i_ were comparable with those calculated from the EPSCs of the climbing and parallel fibre synapses to the Purkinje cell, (CF) and (PF), respectively^27^ (Fig. 4a–d and insets). The former act as low band-pass filters for STD, while the latter acts as high band-pass filters for STF under HFS. Because neural systems usually receive signal packages composed of stimulations at various frequencies^16^ (Fig. 4e inset), we randomly selected several inputs to examine the device responses, as shown in Fig. 4e. The resulting weight modifications varied in the same trend as those in Fig. 4a–d and were comparable with the STD and STF of bio-synapses (Fig. 4e inset). This suggested that either the charging or discharging peaks of the Ba(CF_3_SO_3_)_2_-doped PEO film could be used to mimic the STP of bio-synapses.

We tested 200 film devices; 95% showed the abovementioned electrical property. Although the level of the weight modification phenomenon was affected by environmental factors such as noise and moisture, testing the devices in approximately the same time period permitted negligible environmental variations. The test results were nearly identical both among different devices and across multiple tests of a single device (Fig. 4e), reflecting the intrinsic properties of the materials and devices.

### The simulation of STD and STF

According to the electronic analogies in Figs 3 and 4, we applied the biological EPSC model of STP^27^ (equation (1) and (2)) to our device to examine the analogies in ionic kinetics and establish a microscopic basis for mimicking STP. In neural systems, the signal transmission process is both achieved and affected by the state of the pre-synapse, consisting of the number of effective release sites and the Ca^2+^ flux^15^,^16^,^18^,^27^,^37^. Once the pre-synapse gives trains of stimulations, the Ca^2+^ flux generates numbers of release-ready sites from among the effective sites, thus releasing neurotransmitters and evoking the EPSCs of the post-synapse. The release-ready sites become transitionally ineffective after the release process^15^,^16^,^27^. During the stimulus interval, the ineffective release sites recover effectiveness and the Ca^2+^ concentration in the pre-synapse decays^15^,^16^,^27^. At the moment, the quantity levels are co-decisive to the EPSC variation forms of STP: STD, STF, and the intermediate state in between^15^,^16^.

There are common points between our D_*i*_/F_*i*_ sequences in the Ba(CF_3_SO_3_)_2_-doped PEO electrolyte film and the STD/STF phenomenon in the bio-synapse: both are influenced by the recovery (ENR/ineffective release sites) and decay (IPR/Ca^2+^) levels in the stimulus interval. Considering CF, its high release ability makes the recovery level of ineffective release sites dominate the state of the pre-synapse^16^. The effective release sites in the pre-synapse could be simulated as ENR in the device, with EPSCs simulated as D_*i*_. Accordingly, under LFS, the ineffective release sites remaining after release are fully recovered during sufficient stimulus interval and thus able to generate an EPSC of the same level in response to the next stimulus^15^,^16^,^18^,^27^, which resembles the fully-recovered ENR generating a constant D_*i*_. Meanwhile, the incomplete recovery level of the ineffective release sites, because the HFS has insufficient stimulus intervals, creates fewer effective release sites to release and generate smaller EPSCs, or weight-decreased STD^15^,^16^,^18^,^27^. This resembles the partially-recovered ENR generating decreasing D_*i*_ magnitudes in sequence.

On the other hand, considering PF, its low release ability causes the decay level of Ca^2+^to dominate the state of the pre-synapse^16^. Ca^2+^, or the release-ready sites yielded by Ca^2+^, in the pre-synapse could be modelled by IPR in the device, with EPSCs regarded as F_*i*_. Under LFS, the amount of Ca^2+^ in the pre-synapse is fully decayed during sufficient stimulus interval, thus maintaining the same value after the Ca^2+^ influx triggered by the next stimulus is added. Hence, the same numbers of release-ready sites are yielded by this amount of Ca^2+^, releasing and evoking EPSCs of the samevalue^15^,^16^,^18^,^27^. This process resembles the fully decayed IPR generating constant F_*i*_. Inversely, the incomplete decay level caused by the insufficient stimulus interval in HFS provides additional Ca^2+^ after the next stimulus-triggered Ca^2+^ influx. More release-ready sites are yielded to release, thus generating larger EPSCs, namely weight-increased STF^15^,^16^,^18^,^27^. This process resembles the enhanced IPR based on the partially decayed region generating increasing F_*i*_ values in sequence.

The 995 ms pulse interval of the 1 Hz pulse train in our device, which permitted the full recovery of ionic distribution in the PEO matrix, was parallel to the persistence ability of STP within hundreds of milliseconds^15^. Referring to the biological EPSC model of STP^15^,^16^:


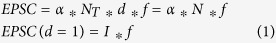



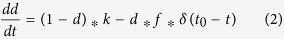


we constructed the weight expressions of D_*i*_ and F_*i*_ sequences as below:


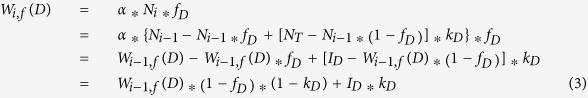



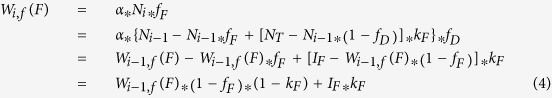


Briefly, because the biological EPSC equations (1) and (2) possess history-based features, the derivative equations (3) and (4) are recursive functions for D_*i*_ and F_*i*_. The character_*i*_stic parameters *f*_*D*_ and *k*_*D*_ are the shrinking and recovery ratios, respectively, of ENR, which correspond to the *f* (release ratio) and *k (*recovery ratio) of effective release sites. *I*_*D*_ is, on behalf of the weight of D_*i*_, generated by the max_*i*_mum ENR, with *I,* on behalf of the EPSC, generated by the maximum effective release sites. The characteristic parameters *f*_*F*_ and *k*_*D*_ are the reduction and enhancement ratios, respectively of the IPR, corresponding to *f* (decay ratio) and *k* (influx ratio) of Ca^2+^-caused release-ready sites (Ca^2+^). *I*_*F*_ is, on behalf of the weight of F_*i*_, generated by the max_*i*_mum IPR, while *I* is generated by the maximum number of Ca^2+^-caused release-ready sites on behalf of the EPSC. The details of the biological significance contrasted with the parameters are shown in Table 2^15^,^16^,^27^. Among the parameters, *f*_*D*_, *k*_*D*_, *f*_*F*_, and *k*_*D*_ are in the range of (0,1] and are functions of frequency; D_1_ and F_1_ are set as 1; *I*_*D*_ = 1; *I*_*F*_ > 1;D_*i*_ diverges from *I*_*D*_and F_*i*_ approaches *I*_*F*_ (Fig. 4 inset)^15^,^16^,^27^. The values of the parameters *f*_*D*_, *k*_*D*_, *f*_*F*_, *k*_*D*_, and *I*_*F*_ were obtained by *f*itting equations (3) and (4) to the D_*i*_ and F_*i*_ sequences in Fig. 4a,c (Table 3), wh_*i*_ch were affected by the salt type and salt concentration in the PEO-based electrolyte film, the thickness of the film, and the depth to which the test probe was inserted, because of the elasticity of the PEO matrix.

**Figure 5:**
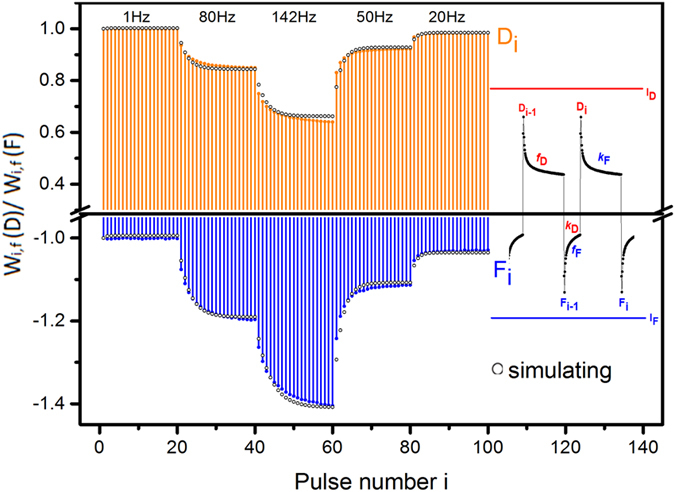
The simulating result of Fig. 4e; the inset shows the parameters in the weight expressions of the D_*i*_and F_*i*_ sequences (equations (3) and (4)).

The variation trends of *f*_*D*_ and *f*_*F*_ were comparable with the biological parameter *f*. It appeared that higher pulse frequencies correlated to larger *f*_*D*_ values. This agreed with CF’s high release ability (*f*) of the effective release sites that caused STD, which grew and acted more intensively at stronger HFS^15^,^16^,^27^, as shown in Fig. 4b and the inset. On the contrary, higher pulse frequencies correlated to smaller values of *f*_*F*_, which also agreed with PF’s low release ability (*f*), namely the low reduction degree of Ca^2+^-caused release-ready sites, which decreased and caused more intensive STF in stronger HFS^15^,^16^,^27^, as shown in Fig. 4d and the inset. Based on the characteristic parameters in Table 3, the simulating results of equations (3) and (4) agreed well with the *W*_*i,f*_ *(D)* and *W*_*i,f*_ *(F)* in Fig. 4e as shown in Fig. 5, supporting not only the fitting result but the feasibility of simulating STP with the Ba(CF_3_SO_3_)_2_-doped PEO electrolyte film device.

Notably, according to the unidirectional signal transmission in neural systems, the D_*i*_ and F_*i*_ of the single device unit cannot be seen as EPSCs simultaneously because they have opposite peak directions. Thus, in neural system-like device integrations containing several elemental device units (or simulated synapses), only the response peaks sharing peak directions can be regarded as single simulation systems. With this limitation, our device can only simulate the interactions of STPs or STDs themselves, rather than those in between, as the bio-synapse network does. Thus, to broaden the future applications of device integration, other devices which conversely manifest STF in charging peaks and STD in discharging peaks must be designed by fabricating different materials into layered structures^5^,^7^,^20^,^38^,^39^,^40^. From another perspective, without restricting the peaks to unidirectional EPSCs, the idea of “feedback plasticity” was introduced, which illustrated the process of the post-synapse inversely transmitting signals to the pre-synapse to inhibit the release of release sites^16^. Accordingly, the opposing D_*i*_ and F_*i*_ signals can be applied simultaneously in a single simulation system, on behalf of either EPSCs or feedbacks. For example, F_*i*_ could be regarded as the feedback signals, simulating the depression of EPSCs (D_*i*_). However, as the feedback plasticity phenomenon is not yet well understood biologically, we cannot know if the exact forms of the feedback signals are identical to F_*i*_. Hence, this biological phenomenon must be further studied before being applied to the development of computational models and simulation devices.

## Conclusions

Devices fabricated from Ba(CF_3_SO_3_)_2_-doped PEO film using the phenomenon of internal ionic relaxation-related back-diffusion are feasible materials to simulate STP^15^,^16^,^18^,^27^. The values of the charging and discharging peaks of the pulse responses did not vary with the input number when the pulse frequency was sufficiently low at 1 Hz. However, the peaks decreased or increased gradually, achieving stable values with increased input numbers at increased frequencies. The weight modification, calculated based on these stable values, varied with the input frequency, resulting in the depression and potentiation of the charging and discharging peaks, respectively. According to these electrical properties, we simulated STD and STF of two bio-synapse types, which manifested high band-pass filtering and low band-pass filtering effects, respectively. The simulations were consistent with biological results and corresponding biological parameters were successfully extracted. Our study suggests the possibility of ion-dependent simulations of STP.

## Additional Information

**How to cite this article**: Chang, C. T. *et al.* Simulation of Synaptic Short-term Plasticity using Ba(CF_3_SO_3_)_2_-doped polyethylene oxide electrolyte film. *Sci. Rep.* **6**, 18915; doi: 10.1038/srep18915 (2016).

## Supplementary Material

Supplementary Information

## Figures and Tables

**Table 1:**
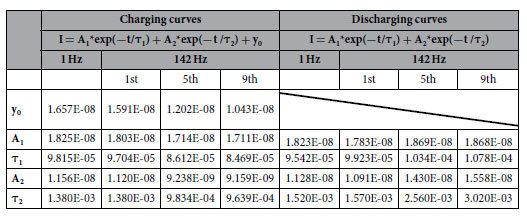
The fitting result of the charging and discharging curves under 1 Hz and 142 Hz.

**Table 2:** The characteristic parameters’ biological significances and correspondences in the PEO-based electrolyte film.

*N*_*T*_	The maximum effective release sites of pre-synapse
	*The initial ENR*
	*The maximum IPR*
*N*	=*N*_T_* d, the number of release-ready sites after previous release/previous Ca^2+^influx
	*The residual ENR*
	*The enhanced IPR*
*d*	The release-ready sites fraction of *N*_*T*_after previous release/previous Ca^2+^influx
*f*	The release fraction/ the decayed Ca^2+^-caused reduction fraction of *N*
*f*_*D*_	*The shrinking fraction of ENR*
*f*_*F*_	*the decay fraction of IPR*
*α*	the average amplitude triggered by releasing an effective release site unit
*I*	=α * *N*_T_* f(*d*=1), the response intensity delivered by the post-synapse when *N*_*T*_ release
*I*_*D*_	*The response intensity delivered in the initial ENR state*
*I*_*f*_	*The response intensity delivered in the maximum IPR state*
*k*	The recovery/the influx Ca^2+^-caused enhancement fraction (toward *N*_*T*_)
*k*_*D*_	*The recovery fraction (toward initial ENR)*
*k*_*F*_	*The enhancement fraction (toward maximum IPR)*
*t*_*0*_	The stimulation moment

**Table 3:** The fitting result obtained from fittingexpressions (3) and (4) to the D_*i*_ and F_*i*_ sequences in Fig. 3a,c.

***f***_***D***_ = 0.00277 + 0.001 * frequency						
***k***_***D***_ = 0.174 + 0.511 * exp(−0.0171 * frequency)						
***f***_***F***_ =−(1 − A * ***k***_***F***_)**/(1 − *k*_*F*_) + 1**						
A = 1.595–0.00333 * frequency						
***k***_***F***_ = 0.213 + 0.319 * exp(−0.0354 * frequency)						
	***I***_***D***_	***f***_***D***_	***k***_***D***_	***I***_***F***_	***f***_***F***_	***k***_***F***_
1 Hz	1	0.0038	0.676	1.582	0.643	0.521
10 Hz		0.013	0.604		0.435	0.437
20 Hz		0.023	0.537		0.310	0.370
40 Hz		0.043	0.432		0.189	0.290
50 Hz		0.053	0.391		0.156	0.267
62.5 Hz		0.065	0.349		0.127	0.248
80 Hz		0.083	0.304		0.099	0.231
100 Hz		0.103	0.266		0.075	0.222
125 Hz		0.128	0.234		0.050	0.216
142 Hz		0.145	0.219		0.034	0.215
