# Supplementary material for: Simulation of synaptic short-term plasticity using Ba(CF3SO3)2-doped polyethylene oxide electrolyte film
Source: Sci Rep. 2016 Jan 7;6:18915. doi: 10.1038/srep18915 (PMC4703968; doi:10.1038/srep18915)
Supplement: Supplementary Information [file srep18915-s1.pdf]

# Supplementary Information

## Simulation of Synaptic Short-term Plasticity using $Ba(CF_3SO_3)_2$-doped polyethylene oxide electrolyte film

C.T. Chang,[1,2] F. Zeng,[1,2] * J.X. Li,[1,2] W. S. Dong,[1,2] S. H. Lu,[1,2] S. Gao,[1] F. Pan[1]*

[1] Key Laboratory of Advanced Materials (MOE), School of Materials Science and Engineering, Tsinghua University, Beijing, 100084, People's Republic of China

[2]Centre for Brain Inspired Computing Research (CBICR), Tsinghua University, Beijing 100084, People's Republic of China

*zengfei@mail.tsinghua.edu.cn, panf@mail.tsinghua.edu.cn

## The thickness the $Ba(CF_3SO_3)_2$-doped PEO film

The $Ba(CF_3SO_3)_2$-doped PEO film was measured about 0.6 μm by SEM (Fig. S1).

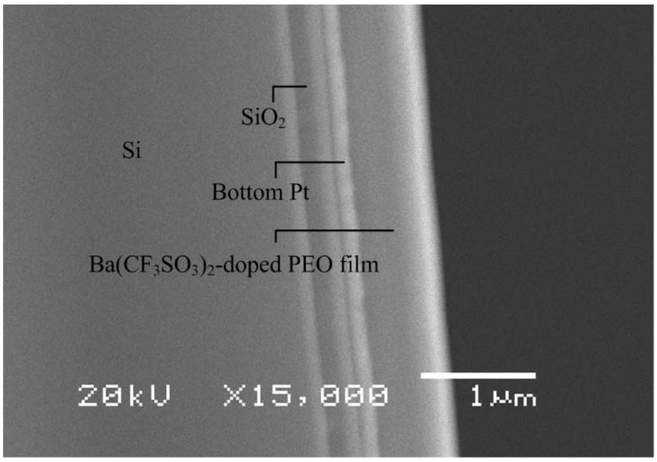


**Figure S1.** TEM image for the thickness measurement.

## The impact of ionic hysteresis and voltage sweeping rate in the DC mode measurement

In the DC mode, both single bias and double bias were measured under multiple voltage sweeping rates: 1, 10, 50, 100 V/s. As shown in Fig. S2, the faster the sweeping rate, the wider the hysteresis loop. Meanwhile, the hysteresis loop saturated under 50V/s and showed nearly identical loop cycles as the ones under 100V/s. It demonstratedthat the ionic hysteresis of$Ba^{2+}$and $CF_3SO_3^-$ inside the PEO matrixacted more intensively during fast voltage sweeping, which provided short time for the ions to respond and migrate.It resulted in thinner polarization layers and thus weaker $E_{in}$ to constrain the current values.

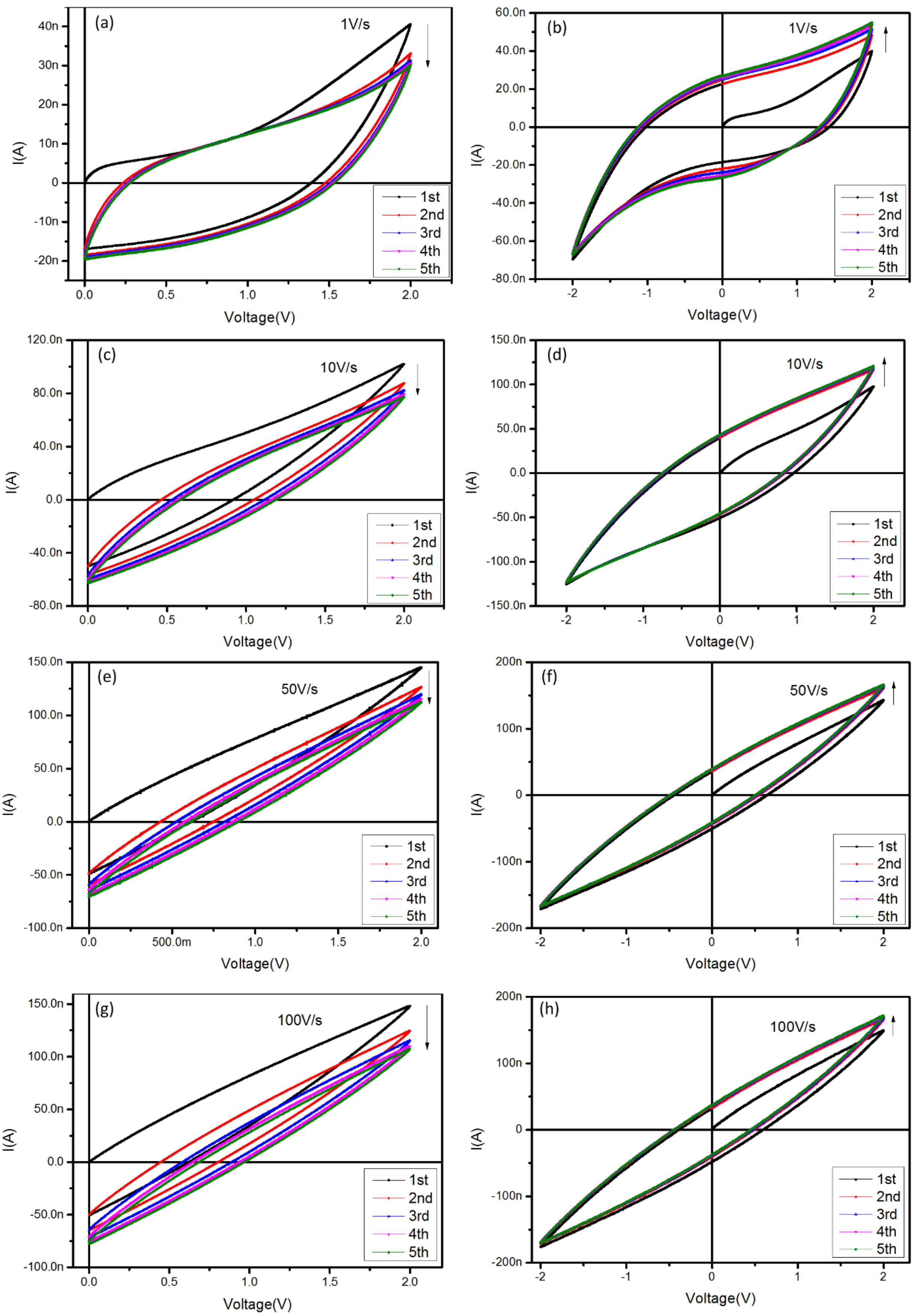


**Figure S2.** Single bias and double bias results measured under multiple voltage sweeping rates: 1, 10, 50, 100 V/s.

(a) Single bias under 1V/s. (b) Double bias under 1 V/s. (c) Single bias under 10 V/s. (d) Double bias under 10 V/s. (e) Single bias under 50V/s. (f) Double bias under 50V/s. (g) Single bias under 100 V/s. (h) Double bias under 100V/s.

## The restoration process under HFS pulse mode

We examined the restoration process of two 142 Hz pulses sequences which were composed of 40 pulses and spaced 60 s to each other. It demonstrated that our device only needed 0.52s to fully restore to the initial state and resulted in same-valued charging/discharging peaks at the second pulse sequence front. The short-term weight modification showed no non-volatile transition related to biological long-term plasticity (LTP).

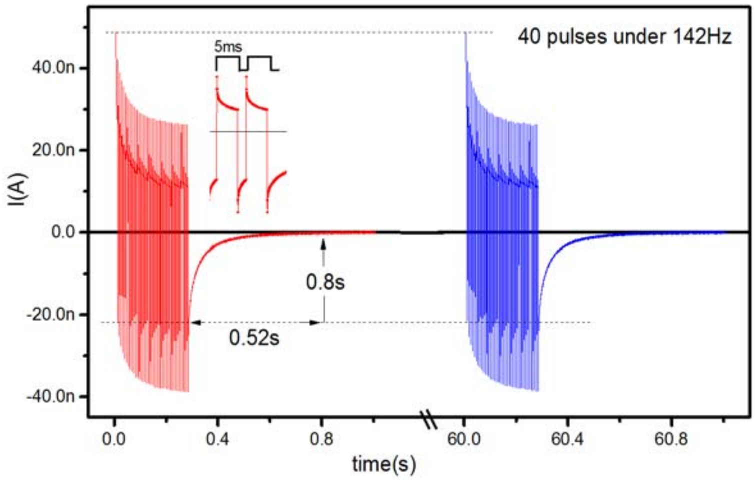


**Figure S3.** The restoration process under HFS pulse mode.

## The responses of the devices functioning general electronic conductance under pulse mode

In our $Ba(CF_3SO_3)_2$-doped PEO film device, the charging and discharging peaks appeared and showed weight modification phenomenon in HFS pulse mode.In order to examine the exact mechanism inside, we tested several electronic conducting devices for comparison. The single resistor, single capacitor, R+C and R//C were testedunder LFS (1Hz) and HFS (142Hz) pulse mode.

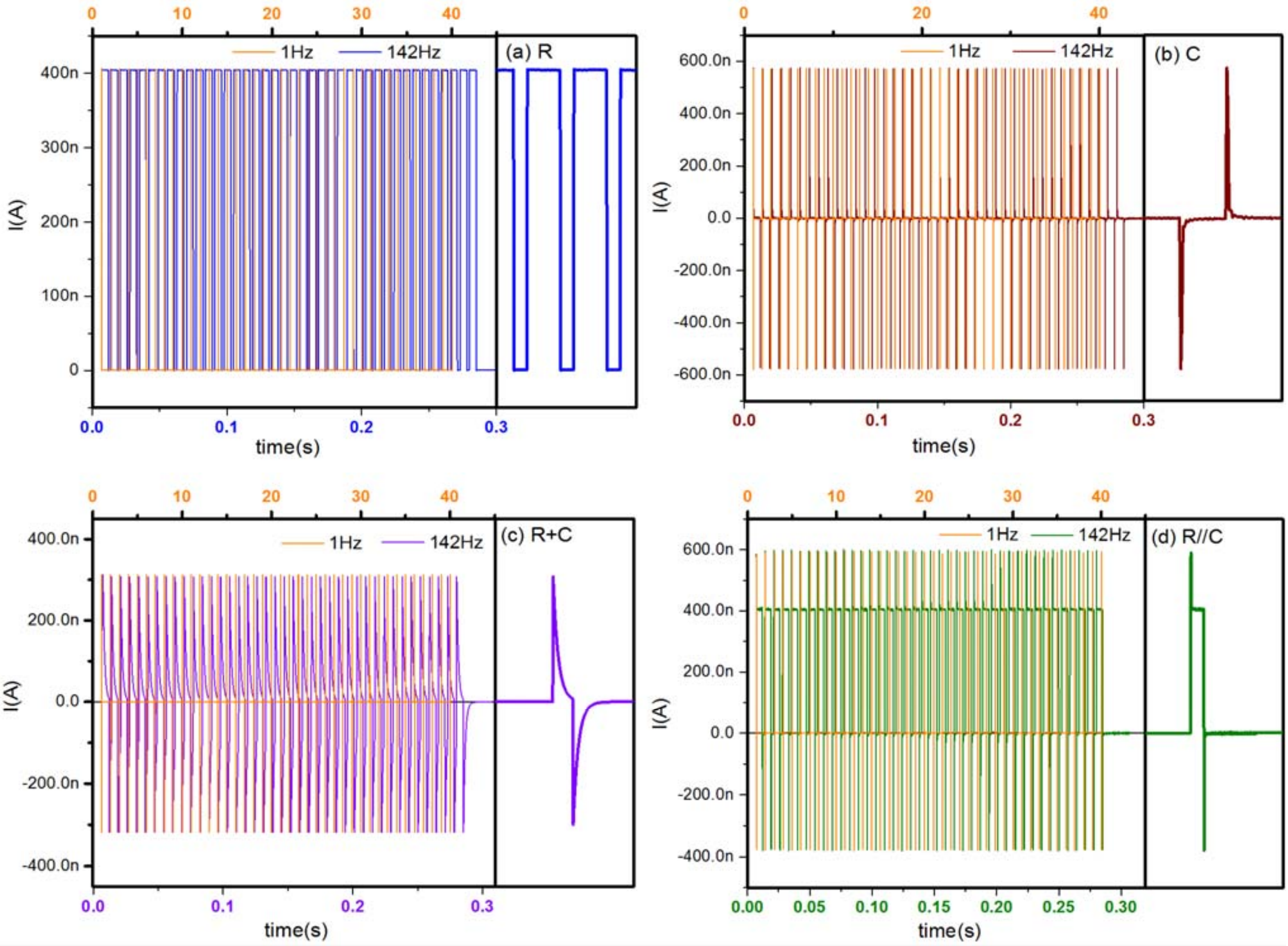


**Figure S4.** The responses of the devices functioning general electronic conductance under 1 Hz and 142 Hz pulse mode. (a) Single resistor.(b) Single capacitor. (c) R+C device. (d)R//C device.

In Fig. S4, apart from the single resistor, all the other electronic conducting devices that consisted capacitor elementshowed both charging and discharging peaks underthe pulse mode.No matter under 1Hz or 142Hz, there was no weight modification foundineither charging or discharging

peaks of the above electronic conducting devices. Meanwhile, the response form of our device was much alike with that of the R//Cdevice. It suggested that the ionic kineticseffect of $Ba^{2+}$ and $CF_3SO_3^-$ in our device gave rise to not only the capacitance characteristics of charge/discharging peaks but additional weight modification phenomenon under HFS, which was not found in the general capacitive devices based onelectronic conducting. The difference between electron's fast response rate to the $E_{ex}$ and ionic hysteresis should be the core of the above phenomenon. It was confirmed in the manuscript by fitting the R//C capacitance equations to the charging and discharging curves of our device.
